# A case of combined 21‐hydroxylase deficiency and CHARGE syndrome featuring micropenis and cryptorchidism

**DOI:** 10.1002/mgg3.730

**Published:** 2019-05-06

**Authors:** Satoko Umino, Miyuki Kitamura, Yuko Katoh‐Fukui, Maki Fukami, Takeshi Usui, Shuichi Yatsuga, Yasutoshi Koga

**Affiliations:** ^1^ Department of Pediatrics and Child Health Kurume University School of Medicine Fukuoka Japan; ^2^ Department of Molecular Endocrinology National Research Institute for Child Health and Development Tokyo Japan; ^3^ Department of Medical Genetics Shizuoka General Hospital Shizuoka Japan; ^4^ National Hospital Organization Kyoto Medical Center Kyoto Japan

**Keywords:** 21‐hydroxylase deficiency, CHARGE syndrome, cryptorchidism, micropenis

## Abstract

**Background:**

21‐hydroxylase deficiency (21‐OHD) is caused due to *CYP21A2* gene variant. In males, the excess androgens produce varying degrees of penile enlargement and small testes. CHARGE syndrome (CS) has a broad spectrum of symptoms. In males, genital features such as micropenis and cryptorchidism are found in 48% of CS. There are no reports of patients with combined 21‐OHD and CS; therefore, it is unknown whether the external genitalia shows penile enlargement or micropenis with/without cryptorchidism.

**Case:**

A boy, born at 37 weeks and 5 days of gestational age with no consanguineous marriage, was admitted to our hospital due to congenital cleft lip, cleft palate, micropenis, cryptorchidism, and a ventricular septal defect. He had severe hyponatremia and hyperkalemia on day 10. He was diagnosed to have 21‐OHD and CS. His external genitalia demonstrated both cryptorchidism and micropenis, but not penile enlargement.

**Methods:**

DNA was extracted from peripheral leukocytes using standard procedures. Sanger sequence was performed in *CYP21A2*. Exome sequence was performed, and then, Sanger sequence was performed around variant in *CHD7*.

**Results:**

Genetic screening for *CYP21A2* gene was performed and compound heterozygous variants of c.293‐13A/C>G (IVS2‐13A/C>G) and c.518T>A (p.I172N) were detected in chromosome 6p21.3. His mother had been heterozygous variant of c.293‐13A/C>G, and his father had been heterozygous variant of c.518T>A. Simultaneously, a *de novo* splicing acceptor alteration in c.7165–4 A>G, in chromodomain helicase DNA binding protein‐7 (*CHD7*), located in chromosome 8q12 was detected, and the patient was diagnosed with 21‐OHD and CS.

**Conclusion:**

Although these two disorders exhibit different modes of inheritance and their co‐morbidity is extremely rare, we encountered one male patient who suffered from both 21‐OHD and CS.

## 1 INTRODUCTION

21‐hydroxylase deficiency (21‐OHD) (OMIM 201910) is caused due to *CYP21A2* gene variant in chromosome 6p21.3 in an autosomal recessive manner. 21‐OHD is further subclassified as follows: (a) salt wasting form (SW), (b) simple virializing form (SV), and the (c) nonclassical form (NC). The symptoms of SV in boys are progressive penile enlargement, small testes, and rapid growth. The symptoms of NC are basically null. The typical symptoms of SW, the most severe type of 21‐OHD, include vomiting, failure to thrive, and skin pigmentation during the neonatal period (Nimkarn, Gangishetti, Yau, & New, 2016). Laboratory findings associated with SW include hyponatremia, hyperkalemia, hypoglycemia, increased adrenocorticotrophic hormone (ACTH), and decreased cortisol. Dysfunction of 21‐hydroxylase in adrenal glands induces excess androgen and 17‐hydroxyprogesterone (17‐OHP) (Nimkarn et al., 2016). In females, excess androgens result in symptoms such as various degrees of clitoral enlargement, labioscrotal fold fusion, and formation of a urogenital sinus. In males, the excess androgens produce varying degrees of penile enlargement and small testes (Nimkarn et al., 2016).

CHARGE syndrome (CS) (OMIM 214800) has a broad spectrum of symptoms, such as coloboma, heart defects, atresia of choanae, retarded growth and development, genital abnormalities, ear anomalies, and/or hearing loss defects (van Ravenswaaij‐Arts & Martin, 2017). The causative gene is mainly chromodomain helicase DNA binding protein‐7 (*CHD7*) located in 8q12.1. CS usually occurs in an autosomal dominant manner. Family histories of CS are rare, and 97% of *CHD7* variants are *de novo* (Sanlaville & Verloes, 2007). In males, genital features such as micropenis and cryptorchidism were found in 48% of CS (Shoji et al., 2014).

To our knowledge, there are no reports of patients with combined 21‐OHD and CS; therefore, it is unknown whether the external genitalia show penile enlargement or micropenis with/without cryptorchidism. Although these two disorders exhibit different modes of inheritance and their comorbidity is extremely rare, we encountered one male patient who suffered from genetic conditions of both 21‐OHD and CS.

### 1.1 Case report

**Figure 1:**
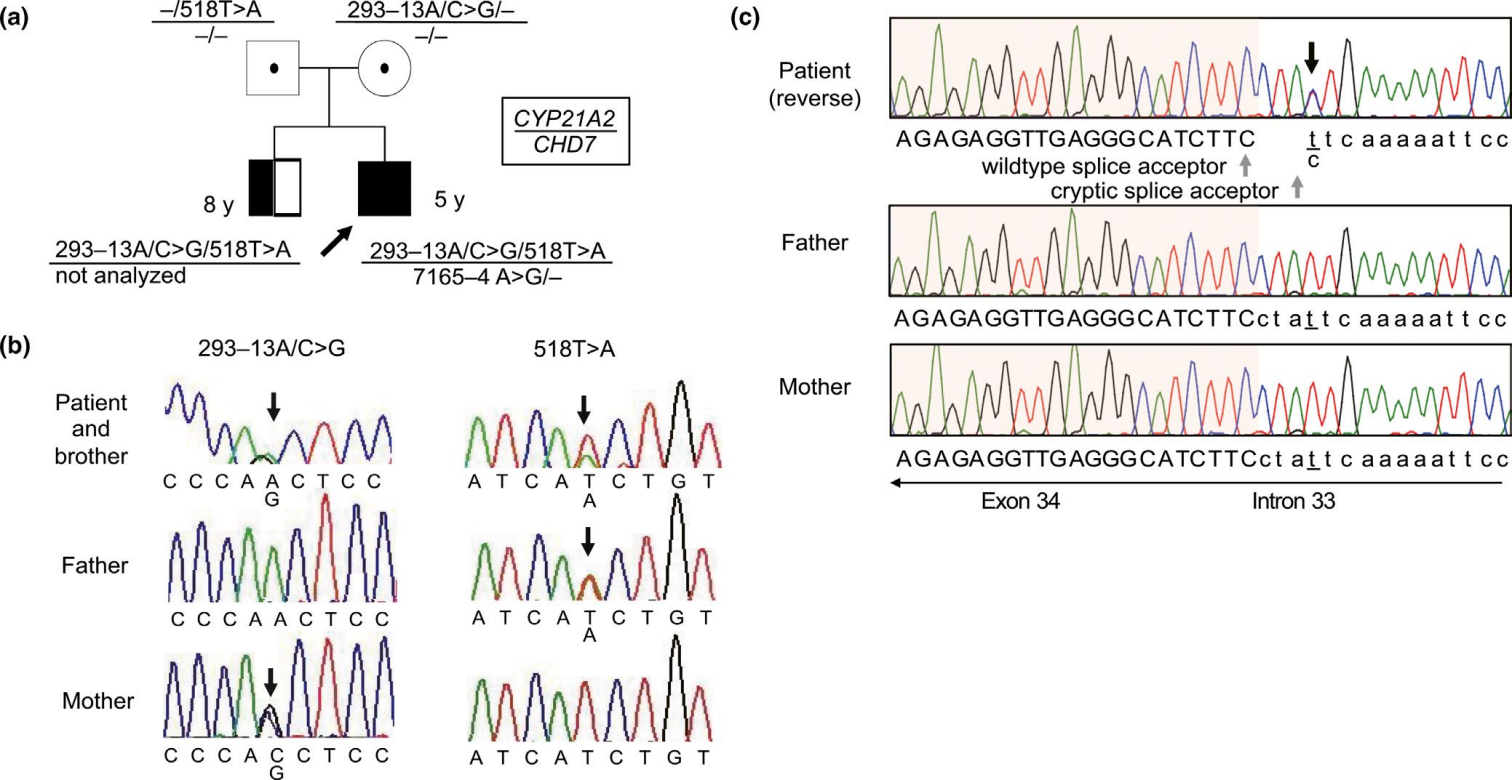
(a) Family tree and variants of *CYP21A2*. The proband and his elder brother had the same compound heterozygous variants of c.293‐13A/C>G/c.518T>A in *CYP21A2*. (b) The compound heterozygous variants of *CYP21A2*. (c) The variants of *CHD7* found in the genomic DNA obtained from the leukocyte of the proband (upper). The shaded area in the chromatogram is exon 34 of wildtype *CHD7*. The three white letters highlighted in green indicate the stop codon in cryptic transcripts (Katoh‐Fukui et al., 2018). The variant was not detected in the parents (middle and lower panel)

The male patient was born at 37 weeks and 5 days of gestation to nonconsanguineous healthy parents. His birth weight was 2,712 g (−0.45 *SD*) and birth height was 46.8 cm (−0.52 *SD*). At the time of birth, a cleft lip, cleft palate, micropenis, cryptorchidism, and ventricular septal defect (VSD) were detected in the patient. Seven days after birth, heart failure developed due to VSD, and diuretic agents were started; furthermore, the patient exhibited severe hyponatremia, hyperkalemia, and hypoglycemia. Neonatal mass screening revealed that his 17‐OHP was elevated to 8.9 ng/ml (reference value <3.5 ng/ml) at 1 day after birth and 18.3 ng/ml at 5 days after birth. He was clinically diagnosed with 21‐OHD. Because his elder brother had previously been diagnosed with 21‐OHD, with compound heterozygous variants of c.293‐13A/C>G (IVS2‐13A/C>G) and c.518T>A (p.I172N) in *CYP21A2* (Figure 1a), genetic screening was performed for this newborn. His mother had been heterozygous variant of c.293‐13A/C>G, and his father had been heterozygous variant of c.518T>A. Hydrocortisone and fludrocortisone were started for 21‐OHD due to hyponatremia and hypokalemia on day 10. At that time, his ACTH level was 67.8 pg/ml and his cortisol level was not measured. He failed to pass an automated auditory brainstem response (AABR) test for both ears on day 7; however, advanced examination of his ears was not performed due to his generally unstable condition. At 2 months of age, he underwent VSD closure, foramen ovale closure, and arterial ligation. Thereafter, his general condition stabilized. For genetic diagnosis of 21‐OHD, DNA was extracted from peripheral leukocytes using standard procedures. Samples were subjected to Sanger sequencing that showed compound heterozygous variants of c.293‐13A/C>G and c.518T>A in *CYP21A2*, same as his elder brother (Figure 1b). He was referred to our hospital at age 3 months due to a change of residence. Bronchoscopy was performed at age 7 months, and showed a flattened epiglottis, pharyngeal softening, and split laryngeal softening. AABR was performed again at age 4 months, and showed bilateral hearing loss, therefore he started using a hearing aid and his bilateral ear anomaly was detected from 6 months of age. He was discharged from our hospital at 6 months. Then, he was repeatedly admitted to our hospital due to respiratory infections and endocarditis. Oral ingestion was difficult due to his flattened epiglottis, pharyngeal softening, and split laryngeal softening. Therefore, nasal feedings were needed. He frequently developed aspiration pneumonia due to gastro‐esophageal reflux. At 1 year of age, he underwent a Nissen's fundoplication. At 15 months of age, his height was −2.0 *SD* and weight was −1.3 *SD*, resulting in a diagnosis of short stature. At 2 years of age, he underwent cryptorchidism. Developmental delays were observed and included standing with help at the age of 15 months and walking alone at 18 months. Cleft palate, micropenis, cryptorchidism, VSD, and bilateral hearing loss indicated CS. The genetic test for CS was performed at 5 years old. Exome sequence was performed, and then, Sanger sequence was performed around variant in *CHD7*. He was diagnosed as CS with a *de novo* splicing acceptor alteration (NM017780.3:c.7165–4 A>G) (Katoh‐Fukui et al., 2018) (Figure 1c). Of note, our patient was diagnosed with *two* novel gene variants of 21‐OHD and CS.

This study was approved by the Institutional Review Board Committees of the National Research Institute for Child Health and Development, National Hospital Organization Kyoto Medical Center, and Kurume University. Written and informed consent was obtained from the family of the patient before reporting this case.

## 2 DISCUSSION

Our case was genetically diagnosed with 21‐OHD and CS. In Japan, the prevalence of 21‐OHD is 1 in 18,000 (Tsuji et al., 2015), and the prevalence of CS is at least 1 in 10,000 (Issekutz, Graham, Prasad, Smith, & Blake, 2005). The likelihood of a patient having both diseases is extremely rare. The relevance of these two diseases is unknown.

According to previous reports of comorbidities associated with 21‐OHD, only two females were reported with Turner's syndrome (Larizza et al., 1994; Montemayor‐Jauregui, Ulloa‐Gregori, & Flores‐Briseno, 1985) and ornithine transcarbamylase (OTC) deficiency (Kim et al., 2013). In both cases, the relevance of the complicated disease was also unknown. On the other hand, according to previous reports of diseases that can coincide with CS, there was one male case with micropenis who was reported as demonstrating Marfan syndrome with an FBN1 gene variant (Chiu, Thakuria, & Agrawal, 2010). Combinations of either 21‐OHD or CS and other genetic or chromosomal diseases are thought to be rare.

Male patients with 21‐OHD often show increased penis length due to excess adrenal androgen exposure. (El‐Maouche, Arlt, & Merke, 2017) Meanwhile, cryptorchidism and micropenis are often observed in patients with CS because of insufficient gonadotropin hormones and androgen (Wheeler, Quigley, Sadeghi‐Nenad, & Weaver, 2000). The patient suffering from both 21‐OHD and CS and showed cryptorchidism and micropenis. These findings indicate that the androgen level after 10 weeks of gestation was presumably a less‐than‐normal level, even though he suffered from 21‐OHD. Similar case reports are required to validate these observations, and female cases with both 21‐OHD and CS may offer interesting examples of how external genitalia can be affected.

Three forms of 21‐OHD are SW, SV, and NC. We diagnosed this case as SW from the symptoms of hyponatremia, hyperkalemia, hypoglycemia, increased ACTH. The poor feeding, weight loss, failure to thrive, vomiting, dehydration, and hypotension might not be shown due to cardiac failure and treatment of intravenous infusion, diuretic medication, hydrocortisone, and fludrocortisone. Penile enlargement was not seen. The symptoms of CS are coloboma, heart defects, atresia of choanae, retarded growth and development, genital abnormalities, ear anomalies, and/or hearing loss defects, which were fully shown in this case. This case had symptoms of both 21‐OHD and CS; however, micropenis and cryptorchidism appeared as the feature of CS.

## 3 CONCLUSION

It is extremely rare to observe overlapping genetically rare diseases, 21‐OHD and CS, in the same individual. For this case, the fetal androgen level may have been less‐than‐normal, although he suffered from 21‐OHD. Untypical clinical features resulted in a definitive diagnosis, which hopefully will benefit patients and families during the treatment and follow‐up periods.

## CONFLICTS OF INTEREST

None declared.
